# Characterizing transverse coherence of an ultra-intense focused X-ray free-electron laser by an extended Young’s experiment

**DOI:** 10.1107/S2052252515015523

**Published:** 2015-09-22

**Authors:** Ichiro Inoue, Kensuke Tono, Yasumasa Joti, Takashi Kameshima, Kanade Ogawa, Yuya Shinohara, Yoshiyuki Amemiya, Makina Yabashi

**Affiliations:** a Department of Advanced Materials Science, Graduate School of Frontier Sciences, The University of Tokyo, 5-1-5 Kashiwanoha, Kashiwa, Chiba 277-8561, Japan; b RIKEN SPring-8 Center, 1-1-1 Kouto, Sayo, Hyogo 679-5148, Japan; c Japan Synchrotron Radiation Research Institute, 1-1-1 Kouto, Sayo, Hyogo 679-5198, Japan

**Keywords:** X-ray free-electron lasers, transverse coherence, beam diagnostics

## Abstract

Characterization of transverse coherence is one of the most critical themes for advanced X-ray sources and their applications in many fields of science. However, for hard X-ray free-electron laser (XFEL) sources there is very little knowledge available on their transverse coherence characteristics, despite their extreme importance. This is because the unique characteristics of the sources, such as the ultra-intense nature of XFEL radiation and the shot-by-shot fluctuations in the intensity distribution, make it difficult to apply conventional techniques. Here, an extended Young’s interference experiment using a stream of bimodal gold particles is shown to achieve a direct measurement of the modulus of the complex degree of coherence of XFEL pulses. The use of interference patterns from two differently sized particles enables analysis of the transverse coherence on a single-shot basis without *a priori* knowledge of the instantaneous intensity ratio at the particles. For a focused X-ray spot as small as 1.8 µm (horizontal) × 1.3 µm (vertical) with an ultrahigh intensity that exceeds 10^18^ W cm^−2^ from the SPring-8 Ångstrom Compact free-electron LAser (SACLA), the coherence lengths were estimated to be 1.7 ± 0.2 µm (horizontal) and 1.3 ± 0.1 µm (vertical). The ratios between the coherence lengths and the focused beam sizes are almost the same in the horizontal and vertical directions, indicating that the transverse coherence properties of unfocused XFEL pulses are isotropic. The experiment presented here enables measurements free from radiation damage and will be readily applicable to the analysis of the transverse coherence of ultra-intense nanometre-sized focused XFEL beams.

## Abstract 2

A new interference technique to measure the transverse coherence of X-ray free-electron lasers is proposed and applied to the characterization of the coherence properties of ultra-intense focused X-ray pulses from the SPring-8 Ångstrom Compact free-electron LAser (SACLA).

## 1. Introduction   

Recent successful operation of X-ray free-electron lasers (XFELs) based on the self-amplified spontaneous emission (SASE) scheme in the hard X-ray region (McNeil & Thompson, 2010 ▸; Emma *et al.*, 2010 ▸, Ishikawa *et al.*, 2012 ▸) enables the use of highly transverse coherent X-rays. In combination with their ultra-intense photon flux and very short pulse duration, XFELs provide novel experimental opportunities in various fields of science (Chapman *et al.*, 2011 ▸; Seibert *et al.*, 2011 ▸; Glover *et al.*, 2012 ▸; Vinko *et al.*, 2012 ▸; Clark *et al.*, 2013 ▸; Tamasaku *et al.*, 2013 ▸, 2014 ▸; Shwartz *et al.*, 2014 ▸). Understanding transverse coherence properties is vitally important for all aspects of XFEL science. First, knowledge of the transverse coherence is essential for designing advanced experiments and analysing complex data. For example, phase retrieval analyses in coherent diffraction imaging (Chapman & Nugent, 2010 ▸) are based on an assumption of fully coherent illumination at the sample. In X-ray photon correlation spectroscopy, excellent transverse coherence is required for achieving a high signal-to-noise ratio with a high speckle contrast (Falus *et al.*, 2006 ▸). Second, information on transverse coherence is necessary for the effective utilization of X-ray optics to control the characteristics of XFEL light. In particular, evaluation of the *M* ^2^ factor, which is a fundamental parameter defining the quality of laser beams and representing the contamination of higher-order transverse coherence modes, is particularly important for achieving a tight focus of XFEL light, because the deviation of *M* ^2^ from the ideal value of unity causes an increase in the size of the focused beam (Yumoto *et al.*, 2013 ▸). Finally, precise measurement of transverse coherence gives useful feedback to accelerator and FEL physics. It is known that the emittance of the electron beam significantly influences the transverse coherence properties (Saldin *et al.*, 2008 ▸, 2010 ▸). Thus, by using transverse coherence as a probe for electron beam diagnostics, we are able to evaluate the electron beam qualities after acceleration and bunch-compression processes to optimize machine parameters. Furthermore, it has been theoretically suggested that the SASE-based XFEL could not have full transverse coherence due to the considerable contributions of the higher-order transverse modes to the total radiation power (Saldin *et al.*, 2008 ▸, 2010 ▸), which is in clear contrast with optical-cavity-based lasers producing a single transverse mode. Precise measurements of transverse coherence enable verification of FEL theory.

Despite its importance, we have very little knowledge of the transverse coherence of hard X-ray FELs due to the difficulty of experimental assessment, especially for focused-beam conditions. The few exceptions are the evaluation of the number of transverse modes by speckle-based techniques (Gutt *et al.*, 2012 ▸; Lee *et al.*, 2013 ▸; Lehmkühler *et al.*, 2014 ▸). A main difficulty of transverse coherence measurement arises from the presence of shot-by-shot fluctuation of the spatial intensity distribution, which originates from the stochastic nature of the SASE-XFEL and the possible instability of the beam pointing. Although transverse coherence of hard X-ray beams from synchrotron X-ray sources has been intensively studied by various kinds of interference techniques (Ishikawa, 1988 ▸; Kohn *et al.*, 2000 ▸; Leitenberger *et al.*, 2001 ▸; Yabashi *et al.*, 2001 ▸; Lin *et al.*, 2003 ▸; Suzuki, 2004 ▸; Pfeiffer *et al.*, 2005 ▸; Snigirev *et al.*, 2009 ▸; Alaimo *et al.*, 2009 ▸; Skopintsev *et al.*, 2014 ▸), these techniques assume or require information on the intensity distributions of the X-ray sources and/or X-ray beams, and thus they are difficult to apply to the characterization of XFEL pulses. For example, the conventional Young’s interference experiment, which determines the transverse coherence of light through the visibility measurement of interference fringes between two beams transmitted through a pair of pinholes or slits, requires knowledge of the intensity ratio at the pinholes (Mandel & Wolf, 1995 ▸). In this case, uncertainty in the intensity ratio causes serious artifacts in the transverse coherence analysis, as pointed out in previous reports on the Young’s experiment at soft X-ray FEL sources (Vartanyants *et al.*, 2011 ▸; Singer *et al.*, 2012 ▸). Another problem arises from the ultra-intense nature of XFEL pulses. Irradiation by an ultra-intense XFEL pulse can easily destroy interferometer devices with just a single-shot exposure. To cope with this issue, one needs to prepare many copies of the interferometer device and align them for each XFEL pulse, although this becomes more difficult as the beam size gets smaller.

To overcome the above difficulties, we propose here a new interference technique, which we call an extended Young’s experiment. The major differences from the original configuration of the Young’s experiment are as follows. First, we use two particles, instead of two pinholes. Second, we employ differently sized particles, rather than identical ones. We apply this scheme to the transverse coherence characterization of focused XFEL pulses from SACLA (Ishikawa *et al.*, 2012 ▸).

## 2. Theory   

We explain the essential points of the scheme as follows. First, we observe interference fringes originating from X-ray beams scattered by two small particles, instead of the pinholes used in the original configuration of the Young’s experiment. By using nanometre-sized gold particles, we can measure the transverse coherence for a spot smaller than a micrometre. Second, instead of manufacturing and aligning many copies of two-particles interferometer devices, we utilize a liquid jet of a suspension of gold particles so as to introduce fresh samples into the interaction region for each XFEL pulse. Importantly, the femtosecond XFEL pulse enables us to record scattering patterns before the particles are destroyed. This scheme, known as a ‘measurement-before-destruction’ framework (Neutze *et al.*, 2000 ▸; Gaffney & Chapman, 2007 ▸), enables us to conduct measurements free from radiation damage, even for ultra-intense X-ray beams. Last, we perform an analysis for each single-shot interference pattern formed by two differently sized particles. In this case, we can determine the transverse coherence on a single-shot basis from a visibility analysis without knowledge of the instantaneous intensity ratio at the two particles, as is shown below. This feature is crucially important for removing the influence of shot-by-shot fluctuations in the spatial intensity distribution on the transverse coherence analysis.

Although SASE-based XFEL light has a high transverse coherence, its longitudinal coherence remains low. Typical durations of XFEL pulses are 10–100 fs, while the longitudinal coherence times are of the order of 0.01 fs. Thus, XFEL pulses consist of a large number of longitudinal modes and can be regarded as quasi-stationary processes. In principle, both transverse and longitudinal coherence properties influence the visibility of the interference fringes. However, we can extract information on the transverse coherence by measuring the visibility in a small-angle scattering geometry. This is because the path-length difference is smaller than the longitudinal coherence length and only the transverse coherence properties are relevant for the degree of visibility.

**Figure 1:**
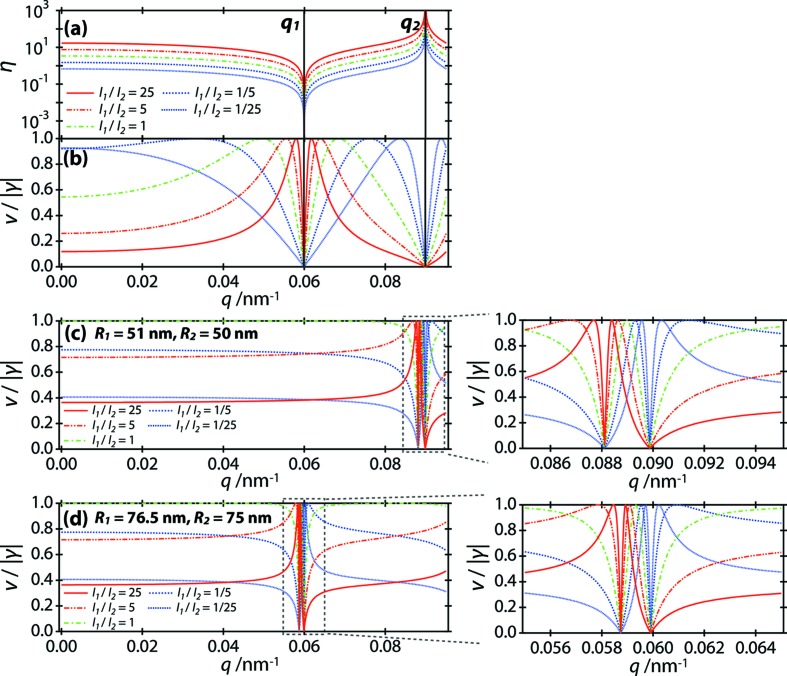
(*a*), (*b*) The dependence of η and the visibility of scattering patterns on the scattering vector *q* for *I*_1_/*I*_2_ = 1/25, 1/5, 1, 5 and 25 for two spherical particles of radii *R*_1_ = 75 nm and *R*_2_ = 50 nm. η(*q*) takes any value greater than or equal to zero, and thus the maximum value of the visibility corresponds to the complex degree of coherence. In part (*a*), *q*_1_ and *q*_2_ satisfy η(*q*_1_) = 0 and η(*q*_2_) = +∞, respectively. For *I*_1_/*I*_2_ = 1/5, 1, 5 and 25, the visibility shows a peak in *q* < *q*_1_. For *I*_1_/*I*_2_ = 1/25, the visibility shows a peak in *q*_1_ < *q* < *q*_2_. For all cases, the first peak value in the visibility along the *q* direction (*q* > 0) corresponds to |γ(**r**_1_, **r**_2_; 0)|. (*c*), (*d*) The dependence of the visibility of scattering patterns on the scattering vector *q* for *I*_1_/*I*_2_ = 1/25, 1/5, 1, 5 and 25 for two spherical particles of similar size [for part (*c*), *R*_1_ = 51 nm and *R*_2_ = 50 nm; for part (*d*), *R*_1_ = 76.5 nm and *R*_2_ = 75 nm].

When a narrow bandwidth XFEL pulse with mean frequency ω irradiates a large-sized particle of radius *R*_1_ at position **r**_1_ and a small particle of radius *R*_2_ at **r**_2_, the intensity of the interference fringes, except for a constant factor, can be expressed as (Mandel & Wolf, 1995 ▸)
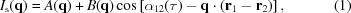
with
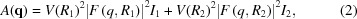
and
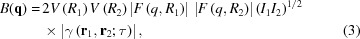
where *I*_1_ and *I*_2_ are the intensities of the incident beam at **r**_1_ and **r**_2_, respectively. In the equations above, *q* is the magnitude of the scattering vector transfer **q** [*q* = (4π/λ)sinθ, where θ is half the scattering angle and λ is the wavelength of the incident radiation], and *F*(*q*, *R*) = 3[sin(*qR*) − *qR*cos(*qR*)]/(*qR*)^3^ and *V*(*R*) are the form factor and the volume of a spherical particle of radius *R*, respectively. τ is the time delay at **q** for the scattering waves originating from the two particles, γ(**r**_1_, **r**_2_; τ) is the complex degree of coherence, and α_12_ is the phase of γ(**r**_1_, **r**_2_; τ). Assuming that the longitudinal coherence time is much longer than τ, |γ(**r**_1_, **r**_2_; τ)| and α_12_(τ) are approximated to be |γ(**r**_1_, **r**_2_; 0)| and α_12_(0), respectively (Mandel & Wolf, 1995 ▸). Then, the visibility of the interference fringes at *q*, *v*(*q*) = *B*(*q*)/*A*(*q*), is given by
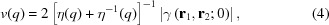
where η(*q*) = *R*_12_(*q*)(*I*_1_/*I*_2_)^1/2^, with 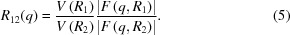
η(*q*) takes any value greater than or equal to zero. One can find certain scattering vectors satisfying η(*q*) = 1, and thus the maximum value of *v*(*q*) equals |γ(**r**_1_, **r**_2_; 0)|. As an example, Fig. 1 ▸(*a*) shows η(*q*) for several values of *I*_1_/*I*_2_ in the case of *R*_1_ = 75 nm and *R*_2_ = 50 nm. Note that η(*q*) is positive infinity when *q* satisfies *F*(*q*, *R*_2_) = 0 (*e.g.* *q* = *q*_2_ in Fig. 1 ▸a), while η(*q*) takes a minimum value of zero when *q* satisfies *F*(*q*, *R*_1_) = 0 (*e.g.* *q* = *q*_1_ in Fig. 1 ▸ *a*). Fig. 1 ▸(*b*) shows the visibility divided by |γ(**r**_1_, **r**_2_; 0)| calculated from equation (4) at the same condition. In every case, the first peak value of *v*(*q*) along *q* (*q* > 0) corresponds to the maximum visibility and equals |γ(**r**_1_, **r**_2_; 0)|. Therefore, by calculating the visibility of the interference fringes for each *q* and finding its first peak along *q*, we can determine the modulus of the complex degree of coherence without *a priori* knowledge of *I*_1_/*I*_2_.

In practice, it is preferable that the particle radii of the two particles are not of similar sizes. As an example, Figs. 1 ▸(*c*) and 1 ▸(*d*) show the visibility divided by |γ(**r**_1_, **r**_2_; 0)| for several values of *I*_1_/*I*_2_ in the case when *R*_1_ is almost the same as *R*_2_ (Fig. 1 ▸ *c*: *R*_1_ = 51 nm, *R*_2_ = 50 nm; Fig. 1 ▸ *d*: *R*_1_ = 76.5 nm, *R*_2_ = 75 nm). Although the first peak value of the visibility along *q* (*q* > 0) corresponds to |γ(**r**_1_, **r**_2_; 0)| for every curve in Figs. 1 ▸(*c*) and 1 ▸(*d*) as in Fig. 1 ▸(*b*), the peak widths of the visibility curves are quite narrow. Thus, one needs to record scattering patterns with high spatial resolutions for a precise determination of |γ(**r**_1_, **r**_2_; 0)|, which may place considerable demands on the experimental setup, especially for X-ray detectors. The visibility takes almost the same value at most scattering vectors except the *q* region in the vicinity of the narrow visibility peaks. As seen from Figs. 1 ▸(*c*) and 1 ▸(*d*), the flat visibility value deviates from |γ(**r**_1_, **r**_2_; 0)| as the intensity mismatch of the incident X-rays at the two particles increases.

## 3. Experiment   

### 3.1. Materials and methods   

Gold spherical colloidal suspensions with nominal radii of 75 and 50 nm (from BBI Solutions) were used for preparing a bimodal colloidal suspension. The polydispersity of the colloidal particles was less than 8% for each suspension. We added water to each suspension, and then mixed the two suspensions. The number densities of colloidal particles of both radii in the mixed suspension were both approximately 1 × 10^9^ particles ml^−1^.

**Figure 2:**
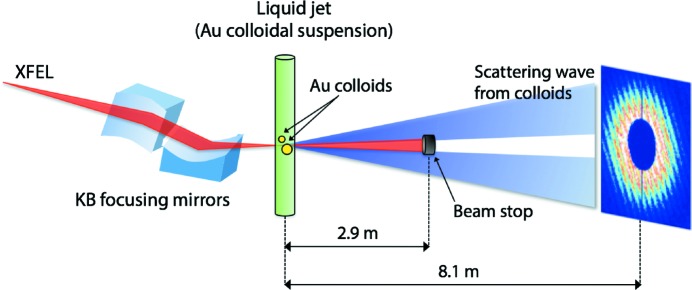
A schematic illustration of the experimental setup for transverse coherence measurement at SACLA. X-ray pulses with a photon energy of 6 keV from SACLA are focused to a size of 1.8 µm (horizontal) × 1.3 µm (vertical) by the KB mirror system. The focused X-ray pulses irradiate a jet of a bimodal colloidal suspension in the MAXIC instrument. The direct beam is blocked by a 4 mm beam stop located 2.9 m downstream of the liquid jet. The MPCCD detector located 8.1 m downstream of the suspension sequentially records the scattering patterns at 30 Hz.

The experimental setup at SACLA beamline BL3 (Ishikawa *et al.*, 2012 ▸; Tono *et al.*, 2013 ▸) for conducting the extended Young’s experiment is illustrated schematically in Fig. 2 ▸. The undulators generate 6 keV XFEL pulses with a pulse energy of 300 ± 40 µJ and a bandwidth of Δ*E*/*E* ≃ 8 × 10^−3^ at 30 Hz. The XFEL pulse contains a few hundred temporal modes in the current operating status of SACLA, which will meet the assumption of a quasi-stationary process of XFEL radiation in equation (1). The X-ray pulses were focused by the focusing mirror system (Yumoto *et al.*, 2013 ▸). The focused beam size measured by the knife-edge scan method was *s*_*x*_ = 1.8 µm (horizontal) × *s*_*y*_ = 1.3 µm (vertical) in full width at half-maximum (FWHM), which corresponds to an ultrahigh intensity that exceeds 10^18^ W cm^−2^. A 7 µm diameter liquid jet of the bimodal colloidal suspension was delivered to the focal point in the multiple-application X-ray imaging chamber (MAXIC) (Song *et al.*, 2014 ▸). We sequentially recorded approximately 400 000 scattering patterns at 30 Hz in a measurement-before-destruction scheme (Neutze *et al.*, 2000 ▸; Gaffney & Chapman, 2007 ▸) with a dual-sensor type MPCCD detector (Kameshima *et al.*, 2014 ▸) located 8.1 m downstream of the focal point. The accessible *q* range, which is determined by the size of the direct-beam stop and the dimensions of the detector, was 0.020 < *q* < 0.13 nm^−1^. In our experimental geometry, the path length differences from the light source to the detector plane *via* two particles are much shorter than the longitudinal coherence length of several tens of nanometres. Thus, |γ(**r**_1_, **r**_2_; τ)| and α_12_(τ) in equation (1) can be approximated to be |γ(**r**_1_, **r**_2_; 0)| and α_12_(0), respectively. Henceforth, we express |γ(**r**_1_, **r**_2_; 0)| as |γ(**r**_1_, **r**_2_)| for simplicity.

### 3.2. Results and discussion   

We extracted interference patterns from the two particles for measurement of the transverse coherence of the X-ray pulses as follows. First, we calculated the total scattering intensity on the detector for each scattering pattern after subtracting the background of the detector. Patterns with a total intensity above a threshold were Fourier-transformed for further analysis. Then, we searched the images for peaks that correspond to interparticle distances, by extracting pixels above a threshold and assuming that neighbouring extracted pixels belong to a common single peak. By counting the number of peaks in the images, we determined the number of irradiated particles. Finally, we were able to extract a set of 324 interference patterns originating from two particles. Although it was recently pointed out that the local wavefront curvatures of XFEL pulses may distort the interference patterns of two particles (Loh *et al.*, 2013 ▸), such an effect was not observed for any of the extracted patterns in the present experiment.

Although the scattering signal from water was quite small compared with that from the two gold particles (the scattering intensity of the two particles was typically about 100 times larger than that from water at each scattering vector *q* used for the subsequent visibility analysis), we eliminated the contribution of the water scattering signal from the measured patterns, as follows. First, we measured the scattering patterns of water in the liquid jet, and calculated the average water scattering pattern and the XFEL pulse energy (*E*_A_). Since the focused XFEL pulses kept almost the same position on the liquid jet, the intensity of the water scattering for each water scattering pattern was almost proportional to the XFEL pulse intensity. Thus, we could estimate the contribution of the water background signal to each extracted pattern of the two particles by multiplying the averaged water scattering pattern by the corresponding XFEL pulse energy normalized by *E*_A_. By subtracting the estimated water background from the extracted patterns of the two particles, we finally obtained scattering patterns that were used for the subsequent visibility analysis.

**Figure 3:**
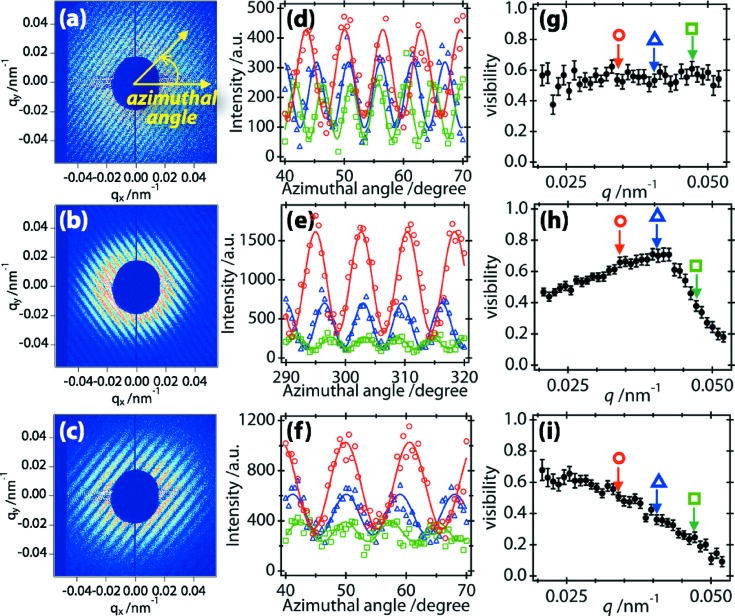
(*a*)–(*c*) Typical scattering patterns from two spherical colloidal particles. (*d*)–(*f*) Intensity profiles along the azimuthal direction at *q* = 0.034 (red circles), 0.041 (blue triangles) and 0.047 nm^−1^ (green squares) in parts (*a*), (*b*) and (*c*), respectively. The curves represent the fitted result with equation (1). (*g*)–(*i*) The *q*-dependence of the visibility of parts (*a*), (*b*) and (*c*), respectively. The arrows represent *q* = 0.034 (red circles), 0.041 (blue triangles) and 0.047 nm^−1^ (green squares), similar to parts (*d*)–(*f*).

Typical extracted patterns after eliminating the water background are shown in Figs. 3 ▸(*a*)–3 ▸(*c*). We analysed the extracted patterns in the region of 0.020 < *q* < 0.055 nm^−1^, as follows. At each *q*, we plotted the intensity profile along the azimuthal direction with an angular range of 30°; this angular range was selected so that the number of interference fringes contained was maximized. The profiles were fitted by equation (1), as shown in Figs. 3 ▸(*d*)–3 ▸(*f*), and the visibility at each *q* and interparticle distance was determined. Figs. 3 ▸(*g*)–3 ▸(*i*) show the visibility for Figs. 3 ▸(*a*)–3 ▸(*c*), respectively. In Fig. 3 ▸(*g*), the visibility is almost constant in the analysed *q* region, indicating that the two irradiated particles are of similar sizes (see Figs. 1 ▸ *c* and 1 ▸ *d*). On the other hand, a clear *q*-dependence of the visibility is measured in Figs. 3 ▸(*h*) and 3 ▸(*i*), due to the different sizes of the two particles. In Fig. 3 ▸(*h*), the visibility shows a clear peak in the measured *q* region. As noted above, this peak value corresponds to |γ(**r**_1_, **r**_2_)|. In Fig. 3 ▸(*i*), the visibility decreases monotonically with *q* in the measured *q* range. This behaviour is the result of *I*_2_ being much larger than *I*_1_. Finally, we extracted 41 patterns that showed a clear peak in the visibility, as seen in Fig. 3 ▸(*h*).

**Figure 4:**
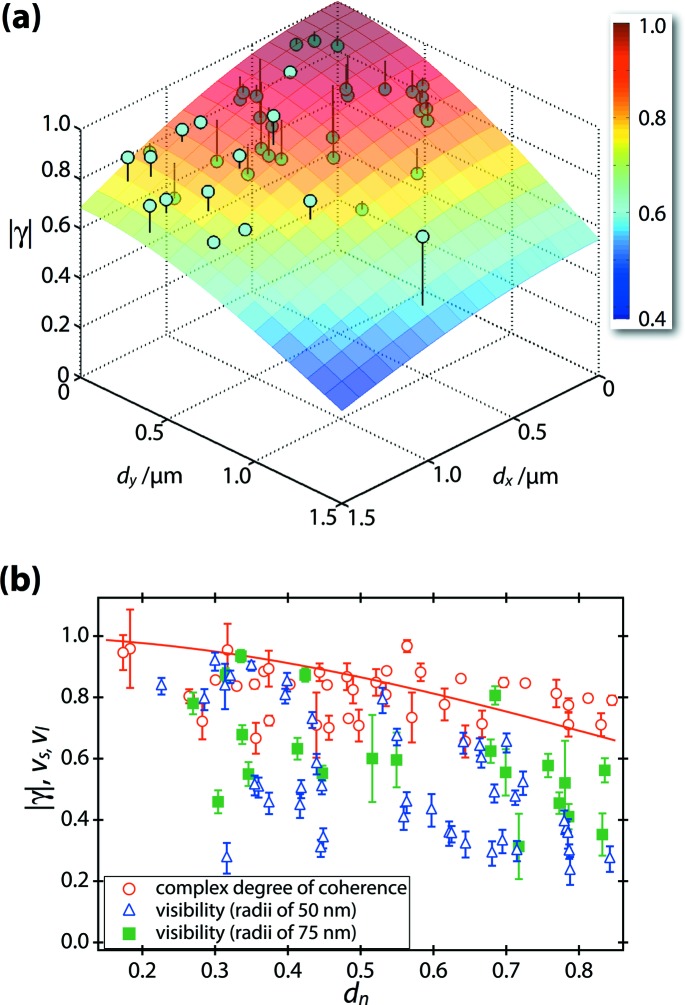
(*a*) A two-dimensional profile of the complex degree of coherence determined with the scattering patterns from two non-identical colloidal particles (blue dots). The surface plot represents the result fitted by a two-dimensional Gaussian function. (*b*) The complex degree of coherence (red circles) and the visibility of the interference fringes originating from two particles with similar radii of ∼50 nm (blue triangles) and ∼75 nm (green squares) as a function of normalized inter-particle distance *d*_n_. The red curve represents a Gaussian fit to the dependence of the complex degree of coherence on *d*_n_.

Using these patterns, we determined the modulus of the complex degree of coherence. Although the complex degree of coherence γ depends on **r**_1_ and **r**_2_ from its definition, we assume that γ can be expressed as functions of interparticle distances in the horizontal (*d_x_*) and vertical (*d_y_*) directions perpendicular to the X-ray beam axis. Here, *d_x_* = |(**r**_1_ − **r**_2_)·**e**_*x*_| and *d_y_* = |(**r**_1_ − **r**_2_)·**e**_*y*_|, where **e**_*x*_ and **e**_*y*_ are the unit vectors in the horizontal and vertical directions perpendicular to the beam axis, respectively. Furthermore, we assume that γ can be represented as a product of the functions *d_x_* and *d_y_*. Fig. 4 ▸(*a*) shows the two-dimensional profile of the transverse coherence, |γ(*d_x_*,*d_y_*)|, determined by the analysis of the interference patterns. A two-dimensional Gaussian fit, 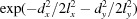, of |γ(*d_x_*,*d_y_*)| provides transverse coherence lengths in the horizontal direction of *l_x_* = 1.7 ± 0.2 µm and in the vertical direction of *l_y_* = 1.3 ± 0.1 µm. As one can see from Fig. 4 ▸(*a*), the function 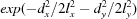 is a reasonable approximation for describing |γ(*d_x_*,*d_y_*)|, which indicates the validity of the above assumptions for γ. Interestingly, the ratios between the coherence lengths and the focused beam sizes are almost the same in the horizontal and vertical directions. Since the intensity profile of the *unfocused* XFEL beam is isotropic (Tono *et al.*, 2013 ▸), the profile of the two-dimensional transverse coherence for the *unfocused* beam is also considered isotropic.

To show explicitly the power of the use of two particles with well separated radii, we also analysed in detail the visibility of the scattering patterns of two particles with similar radii. As seen from Figs. 1 ▸(*c*) and 1 ▸(*d*), the visibility of two particles with similar radii of ∼50 and ∼75 nm maintains an almost constant value in the analysed *q* region of 0.020 < *q* < 0.055 nm^−1^. Based on this feature, we extracted scattering patterns for two particles with similar radii whose visibility is almost constant for 0.020 < *q* < 0.055 nm^−1^, like Fig. 3 ▸(*g*). As the relation *R*_1_ ≃ *R*_2_ holds in this case, *A*(**q**) in equation (1), which is a fitting parameter in the visibility analysis, is proportional to the square of the form factor of the particles [|*F*(*q*, *R*_1_)|^2^ and |*F*(*q*, *R*_2_)|^2^]. Thus, we can know whether the XFEL pulse irradiated two particles with radii of ∼50 or ∼75 nm from the visibility analysis. Fig. 4 ▸(*b*) shows the modulus of the complex degree of coherence |γ| (red circles) determined by the extended Young’s experiment, and the visibility of the interference fringes originating from two small particles (∼50 nm), *v*_s_ (blue triangles), and that originating from two large particles (∼75 nm), *v*_l_ (green squares), as a function of the normalized inter-particle distance *d*_n_ = [(*d_x_*/*s_x_*)^2^ + (*d_y_*/*s_y_*)^2^]^1/2^. Here, *v*_s_ and *v*_l_ were determined by averaging the visibility for 0.020 < *q* < 0.055 nm^−1^ in scattering patterns when the two irradiated particles were of similar sizes. Compared with |γ(*d*_n_)|, significant shot-to-shot fluctuation of *v*_s_ and *v*_l_ was measured at each *d*_n_. This behaviour is due to the mismatch in the intensity ratio at two particles, as seen from Figs. 1 ▸(*c*) and 1 ▸(*d*). What we can determine from *v*_i_ is not the exact value of |γ(*d_x_*, *d_y_*)| but the lower limit. In contrast, we can determine the exact value of |γ(*d_x_*, *d_y_*)| by analysing scattering patterns from two particles with well separated radii.

Finally, we evaluated the total degree of transverse coherence (Saldin *et al.*, 2008 ▸, 2010 ▸), ζ, which is defined as
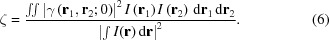
By approximating the beam profile with a two-dimensional Gaussian function having a FWHM of the measured beam size, we estimated ζ to be 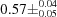 based on equation (6). Since the number of transverse coherence modes is given by 1/ζ for an XFEL operated in the deep nonlinear region (Saldin *et al.*, 2010 ▸), this estimated value of ζ corresponds to 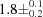 transverse coherence modes, indicating that the 6 keV focused XFEL pulses from SACLA are dominated by only a few transverse modes. This value of the number of transverse coherence modes corresponds perfectly with the previous transverse coherence characterization of the 8 keV XFEL pulses from SACLA by a speckle-based technique (Lehmküehler *et al.*), where the number of transverse coherence modes was estimated to be 1.6 from the statistical properties of the speckle patterns of dense colloidal systems.

## 4. Conclusion   

We have proposed a simple interference experiment to measure the transverse coherence properties of X-ray pulses from XFELs. We applied this experiment to ultra-intense focused X-ray pulses from SACLA and successfully measured the two-dimensional transverse coherence. It should be emphasized that this is the first direct measurement of the profile of two-dimensional transverse coherence with the transverse coherence length of hard XFEL sources. We found that the ratios between the coherence lengths and the focused beam sizes are almost the same in the horizontal and vertical directions. By combining our results and the fact that the beam profile of the *unfocused* XFEL beam is isotropic, it is suggested that the transverse coherence properties of the *unfocused* XFEL beam are also considered to be isotropic.

Finally, we discuss a future perspective. Recent developments in focusing techniques allow the use of nanometre-focused XFEL pulses with a power density of over 10^20^ W cm^−2^ (Mimura *et al.*, 2014 ▸), which provides novel opportunities for the investigation of quantum electrodynamics and high-order nonlinear X-ray effects, as well as imaging of single biological molecules. The transverse coherence of such an ultra-intense beam is an intriguing subject. By using bimodal particles with smaller radii than the focused beam size, the extended Young’s experiment presented here enables measurements free from radiation damage and will readily provide a pathway to approach the transverse coherence of ultra-intense nanometre-sized focused XFEL beams.
